# Supplementary material for: Exposure to perfluorooctanoic acid accelerates Drosophila melanogaster juvenile development and disrupts mitochondrial metabolism
Source: bioRxiv. 2026 Jun 17:2026.06.14.730922. Preprint. [Version 1] doi: 10.64898/2026.06.14.730922 (PMC13307966; doi:10.64898/2026.06.14.730922)
Supplement: Supplement 1 — Figure S1. FOXO subcellular localization following PFOA exposure. Fat bodies were dissected from L2 larvae at 60 h after egg laying (AEL). Tissues were immunostained using an anti-FOXO antibody. (A) Representative images of fat bodies from larvae exposed to 0 μM or 500 μM PFOA, showing DAPI and FOXO channels individually and merged. (B) Quantification of the nuclear-to-cytoplasmic FOXO ratio measured using ImageJ. Each data point represents a field of view from 4–5 fat bodies. Data were analyzed using a Kruskal–Wallis test with multiple comparisons. No significant differences were found. Figure S2. Effects of PFOA exposure on adult wing size under elevated temperature conditions. Oregon R larvae were reared at 29 °C and exposed to increasing concentrations of PFOA. Wing lengths were measured in adult (A) females and (B) males 1 day after eclosion (n=24–40 wings). Data were analyzed using Kruskal–Wallis tests with multiple comparisons to the 0 μM control. *p < 0.05. **p < 0.01. ***p < 0.001. **** p < 0.0001. Figure S3. PFOA exposure has limited effects on the larval metabolome. Semi-targeted metabolomics was performed on whole larvae exposed to 0, 0.036 μM and 3.6 μM PFOA. (A,B) Heatmaps showing the metabolites that exhibited a statistically significant change p < 0.05 following developmental PFOA exposure. Data was normalized to sample mass and the spike-in internal standard. Data analysis conducted with Metaboanalyst 6.0. Metabolites were measured by LC–MS at 84 h AEL using 5 biological replicates per condition, each consisting of 25 larvae. Statistical analyses were conducted using MetaboAnalyst 6.0. Figure S4. Biocalorimetry thermal conductance measurements. (A) Schematic of the sensing capillary channel of the biocalorimeter, indicating the stopper, sensing thermistor, and the position of the larvae during heat output measurements. (B) Dark-field optical microscopy images of the sensor region without a larva and with a single larva positioned near the stopper. ( [file media-1.pdf]

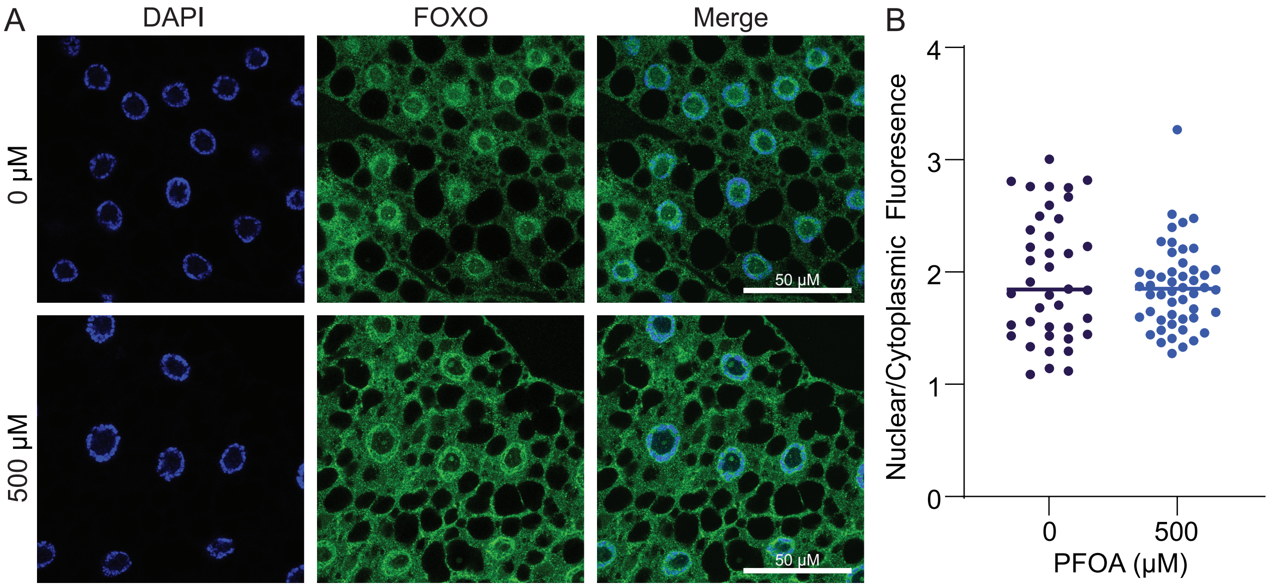

Figure S1. FOXO subcellular localization following PFOA exposure. Fat bodies were dissected from L2 larvae at 60 h after egg laying (AEL). Tissues were immunostained using an anti-FOXO antibody. (A) Representative images of fat bodies from larvae exposed to 0 µM or 500 µM PFOA, showing DAPI and FOXO channels individually and merged. (B) Quantification of the nuclear-to-cytoplasmic FOXO ratio measured using ImageJ. Each data point represents a field of view from 4-5 fat bodies. Data were analyzed using a Kruskal–Wallis test with multiple comparisons. No significant differences were found.

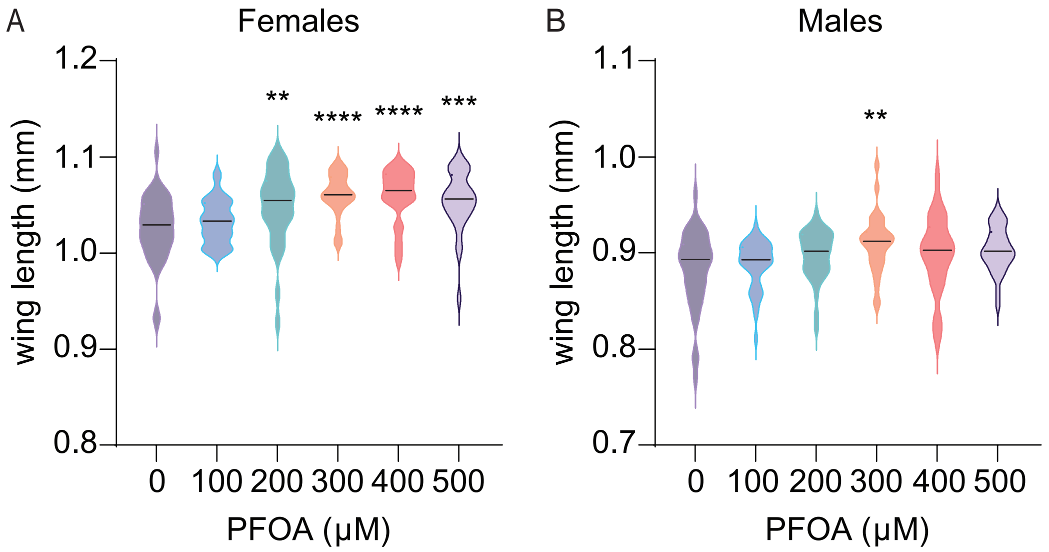

Figure S2. Effects of PFOA exposure on adult wing size under elevated temperature conditions. Oregon R larvae were reared at 29 °C and exposed to increasing concentrations of PFOA. Wing lengths were measured in adult (A) females and (B) males 1 day after eclosion (n=24-40 wings). Data were analyzed using Kruskal–Wallis tests with multiple comparisons to the 0 µM control. *$p < 0.05$. **$p < 0.01$. ***$p < 0.001$. **** $p < 0.0001$.

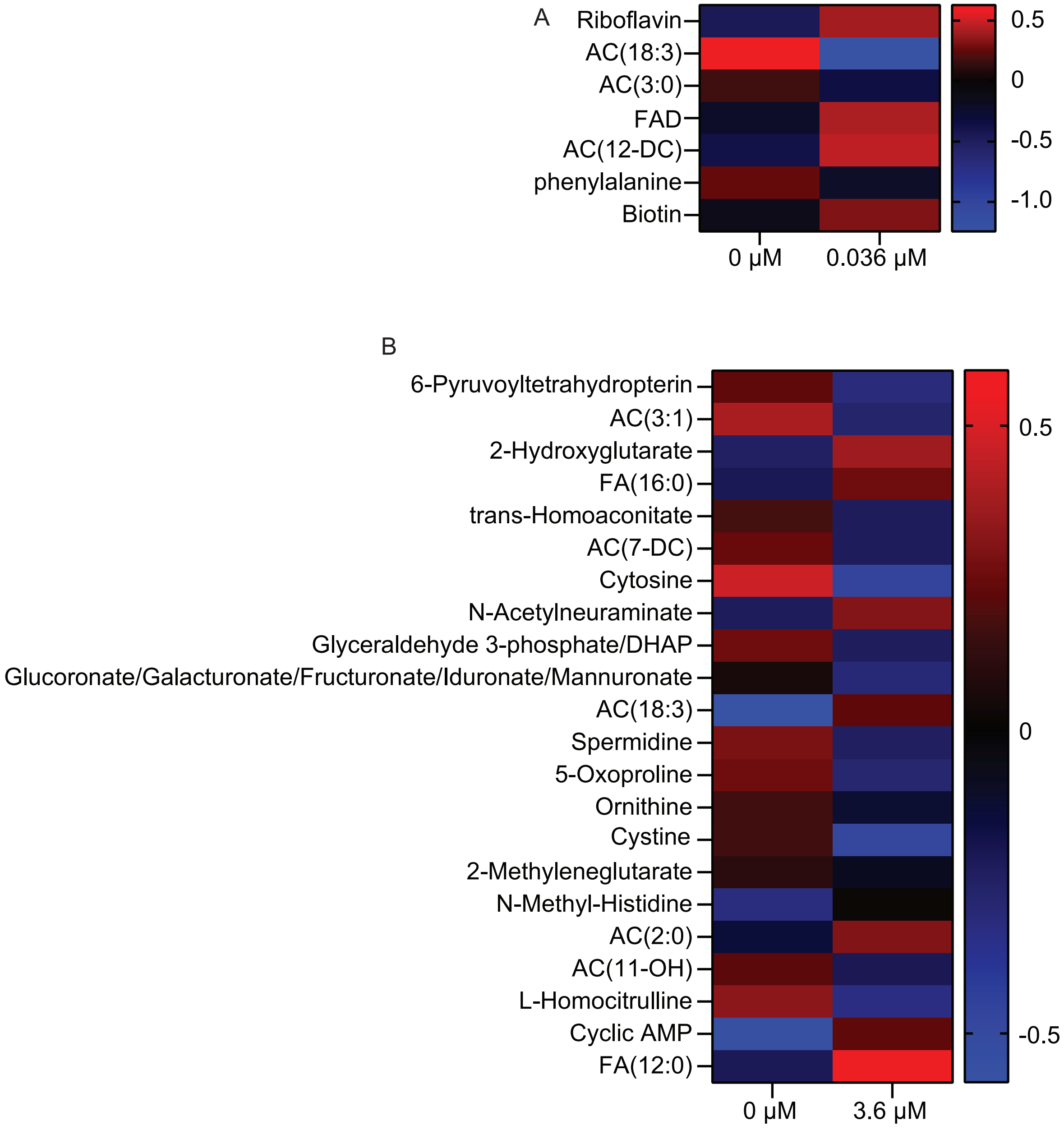

Figure S3. PFOA exposure has limited effects on the larval metabolome. Semi-targeted metabolomics was performed on whole larvae exposed to 0, 0.036 μM and 3.6 μM PFOA. (A,B) Heatmaps showing the metabolites that exhibited a statistically significant change $p < 0.05$ following developmental PFOA exposure. Data was normalized to sample mass and the spike-in internal standard. Data analysis conducted with Metaboanalyst 6.0. Metabolites were measured by LC–MS at 84 h AEL using 5 biological replicates per condition, each consisting of 25 larvae. Statistical analyses were conducted using MetaboAnalyst 6.0.

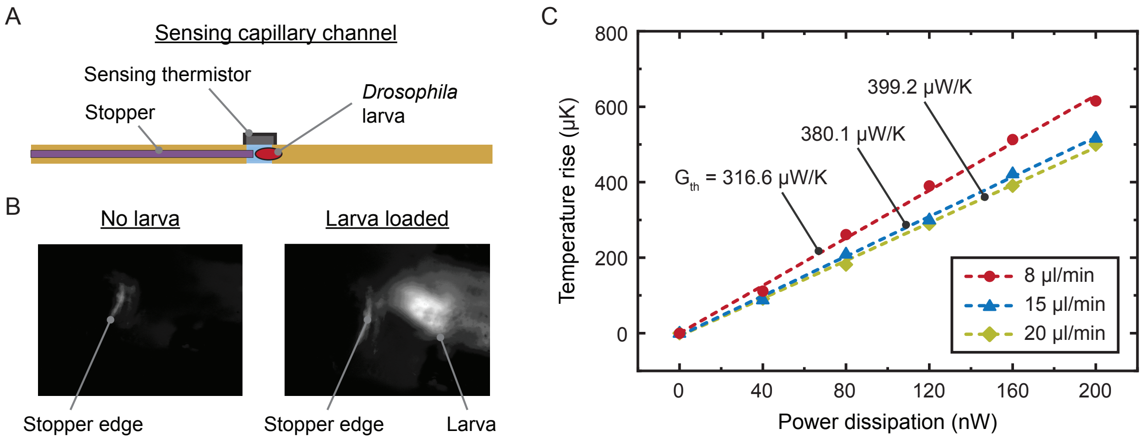

Figure S4. Biocalorimetry thermal conductance measurements. (A) Schematic of the sensing capillary channel of the biocalorimeter, indicating the stopper, sensing thermistor, and the position of the larvae during heat output measurements. (B) Dark-field optical microscopy images of the sensor region without a larva and with a single larva positioned near the stopper. (C) Thermal conductance (G_th) of the sensing capillary at different medium flow rates, measured to be approximately 316.6, 380.1, and 399.2 μW/K at flow rates of 8, 15, and 20 μL/min, respectively.
